# Reconsidering routine admission for children under age 3 undergoing partial tonsillectomy: a prospective study

**DOI:** 10.1186/s40463-023-00659-0

**Published:** 2023-09-23

**Authors:** Ameen Biadsee, Craig Nathanson, Or Dagan, Firas Kassem, Avishai Stahl, Tova Mishali, Yaniv Ebner, Brian Rotenberg

**Affiliations:** 1 Department of Otolaryngology–Head and Neck Surgery, Schulich School of Medicine and Dentistry, St. Joseph Hospital, Western University, B2-501, 268 Grosvenor Street, London, ON N6A 4V2 Canada; 2 Department of Otorhinolaryngology–Head and Neck Surgery, Meir Medical Center, Kfar Saba, Israel; 3 Sackler Faculty of Medicine, Tel Aviv University, Tel Aviv, Israel; 4 Department of Child and Adolescent Psychiatry, Schulich School of Medicine and Dentistry, Western University, London, ON Canada; 5 Department of Dermatology and Venereology, Soroka Medical Center, Beer Sheva, Israel; 6 Department of Pediatric Surgery, Meir Medical Center, Kfar Saba, Israel

**Keywords:** Partial tonsillectomy, Adenoidectomy, Tonsils, Sleep disordered breathing, Micro-debrider, Pediatric length of stay, Inpatient monitoring, Tonsillectomy guidelines

## Abstract

**Background:**

Partial Tonsillectomy (PT) is an alternative method to treat sleep disordered breathing (SDB) and/or obstructive sleep apnea (OSA). The current guidelines do not differentiate it from traditional tonsillectomy. Thus, children younger than 3 years old undergoing PT are admitted for surveillance similar to traditional tonsillectomy due to possible postoperative complications. The aim of this study is to assess the risks of PT in children 3 years old and younger, compared to older children.

**Methods:**

Children underwent inpatient partial tonsillectomy and/or adenoidectomy, due to SDB/OSA, from 2018 to 2020. A special protocol was designed, including follow-up at 2-, 4-, 6-, 8- and 24-h after surgery. Variables analyzed included visual analogue pain score, oral intake, oxygen saturation, pulse rate, postoperative hemorrhage, urine output, temperature, analgesics and fluid administration. Furthermore, major interventions were recorded. Comparison of all variables between children younger than 3 years old with older children was performed.

**Results:**

Ninety-two children were included; mean age of the whole cohort was 44.5 ± 21.9 months. Thirty-five (38%) children were 3-years old or younger and n = 57 (62%) were older than 3 years old, with no significant statistical difference in sex (*p* = 0.22). Mean age in the younger group was 25.7 ± 6.9 months, and 56.1 ± 20.1 months in the older group. In total we had 7 children with post-operative complications; 4 with fever, 3 with low intake. There were no major interventions recorded in either group. The complications were more common in the older group (n = 5) than the younger group (n = 2) without a statistical significance (*p* = 0.59). There were no differences in VAS, use of painkillers, oral intake, urine output, oxygen saturation and tachycardia among the two groups.

**Conclusion:**

This study supports that children undergoing ambulatory PT may be at low risk of complications, regardless of age.

**Graphical abstract:**

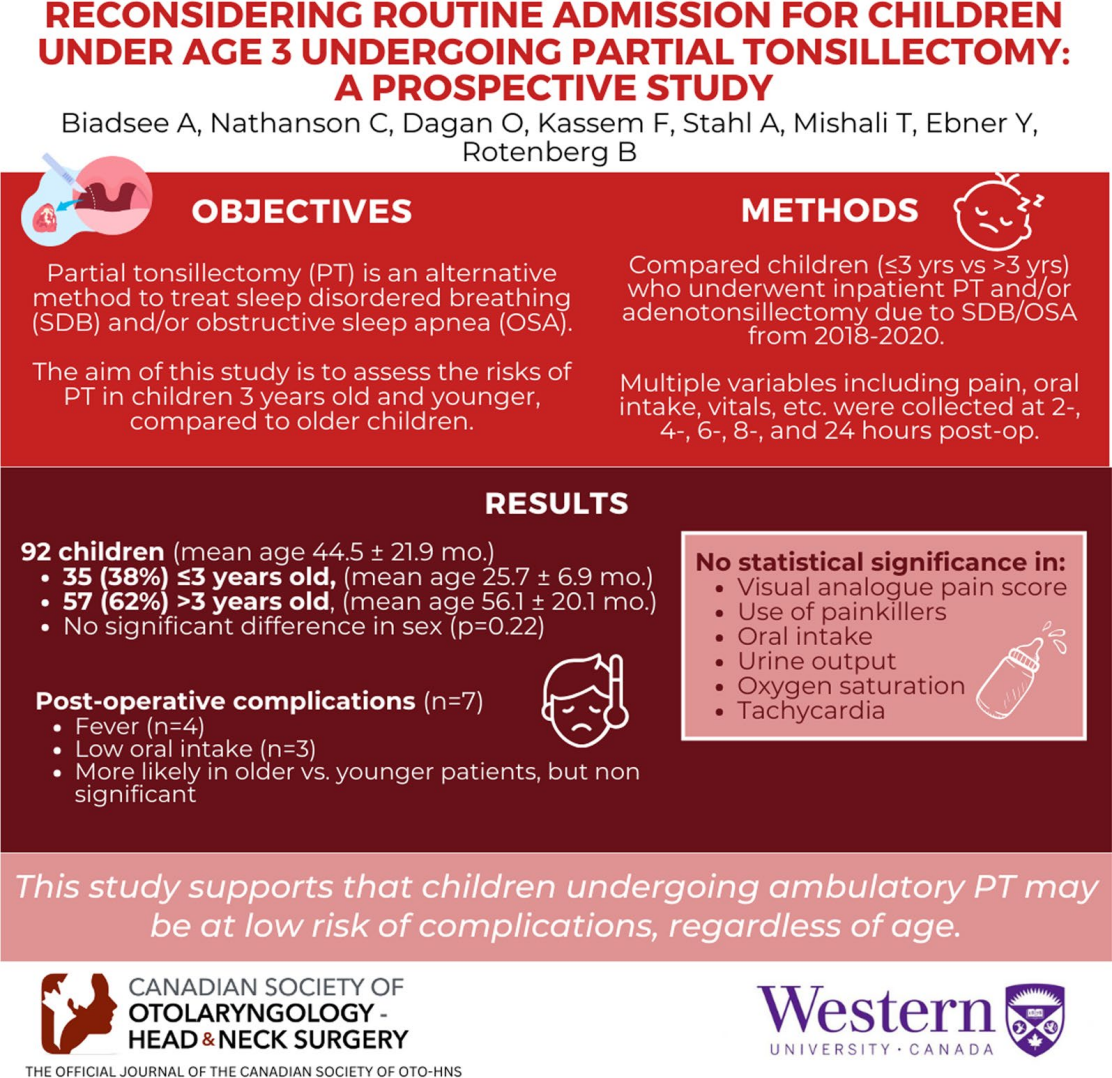

## Introduction

Partial tonsillectomy (PT) is one of the most common operations in pediatric otolaryngology. It is variously described as tonsillotomy, intra-capsular tonsillectomy and subcapsular tonsillectomy. All terms refer to the technique which involves removing most of the tonsil lymphoid tissue, while sparing the capsule [1]. The remaining lymphoid tissue and capsule serve as a protective sheet and as a biological dressing over the pharyngeal muscle layer and underlying vessels, to improve pain and decrease swelling.

In 2019, the American Academy of Otolaryngology published updated clinical practice guidelines for pediatric tonsillectomy [2]. They recommend tonsillectomy for children with sleep disordered breathing (SDB) or obstructive sleep apnea (OSA). However, they did not include information regarding PT, even though this was gaining popularity among otolaryngologists worldwide and is used primarily for treating SDB/OSA.

According to the American guidelines, total tonsillectomy can be performed as an outpatient surgery, however, inpatient monitoring is recommended for children younger than 3-years or with severe OSA [2]. Similar recommendations were stated in the French tonsillectomy guidelines [3]. Of note, neither of the previous guidelines differentiated between tonsillectomy and PT. The 2018 Israeli guidelines were the first to differentiate between the two, recommending that children undergoing PT can be discharged after 6 h, in case of the child is older than 2 years old [4]. However, these guidelines are not yet carried out in daily practice. In reality, in publicly-funded hospitals in Israel children are admitted for 24 h surveillance after surgery, regardless of their age.

The recommendation of inpatient monitoring for young children is supported by previously published studies that showed a high rate of respiratory complications in children younger than 3 years old and weight less than 14 kg, after total tonsillectomy [5–7]. However, two published retrospective studies comparing the safety and outcomes of PT in children younger than 3 years old versus older children, showed no increased post-operative complications in either group [8, 9]. These published cohorts warrant questioning the safety of PT in children younger than 3 years old and differentiating between PT and total tonsillectomy.

The aim of this prospective pilot study is to assess the risks of PT in children 3 years old and younger, compared to older children.

## Materials and methods

This prospective cohort study was conducted in the Department of Otolaryngology, Meir Medical Center, Israel (affiliated with Tel-Aviv University) and approved by the Institutional Review Board (0276-18-MMC).

The study was conducted 2018–2020, and included children 1–12 years-old who underwent powered intracapsular tonsillectomy and adenoidectomy (with or without insertion of ventilation tubes) in our department, due to clinical symptoms of SDB. SDB was diagnosed based on history and physical examination. Children with severe obesity (body mass index for age at the 99th percentile or above), Down syndrome, craniofacial abnormalities, neuromuscular disorders, cyanotic heart diseases or mucopolysaccharidoses were excluded. Parents provided a written informed consent.

### Surgical technique

Procedures were performed under general anesthesia. Enlarged palatine tonsils were partially removed from within the capsule using a straight blade micro-debrider (Medtronic) and adenoid tissue was removed using a curette. Hemostasis was accomplished using local packing with gauze and suction cautery. Surgeries were performed in the morning by residents under the supervision of an attending otolaryngologist. Patients received 0.15 mg/kg intravenous dexamethasone preoperatively.

### Follow-up protocol

All PT procedures performed in our institution are inpatient PT, hence the child is kept for surveillance for at least 24 h post-operatively. Essentially, all children were transferred to PACU for recovery, and then admitted for a 24-h overnight surveillance in the Pediatric Surgery Department.

We built a special follow-up protocol designed for this study which included follow-up at 5 time-points: 2-, 4-, 6-, 8- and 24-h post-surgery. Follow-up at each time-point included: 1. visual analogue pain scale (VAS). Scores were given subjectively by the nurse and the parents. 2. Oral intake: any oral liquid or solid intake was reported by the parents. If a successful intake was recorded in one time-point, the subsequent time-points were considered normal. 3. Oxygen saturation, below 93% was considered abnormal 4. Heart rate between the 10th and 90th age-specific percentiles was considered normal. 5. Physical examination; i.e. to rule out bleeding. Children were examined by a resident for signs of oral or nasal bleeding. 6. Urine output. If the child urinated at any time-point, the subsequent time-points were considered normal. 7. Temperature; Sub-axillary temperature above 37.5 °C was considered elevated. 8. Intake of oral analgesics. 9. Intravenous fluid administration.

From the 8th hour post surgery and onward, a standard inpatient clinical monitoring was performed by the nurses, which included bed-side evaluation every 2–3 h. Since monitoring of SpO2 was not continuous, the team was instructed to closely observe these children and report any change in the clinical status. This monitoring included clinical observation for any apneas, significant snoring, increased work of breathing or bleeding.

After the 24-h follow-up, a clinical decision regarding discharge was made (in consultation with a senior, fellowship-trained, pediatric otolaryngologist (Y.E.)). Children were discharged if they had an optimal oral intake and an optimal respiratory status (normal breathing pattern and without increased work of breathing). Reasons for continued admission were defined a priori as: (1) inadequate oral intake, (2) poor hydration, (3) sub-axillary temperature above 38.5 °C, (4) desaturation, or (5) anuria. Furthermore, any major interventions (such as; re-intubation, oxygen supplementation due to desaturation lower than 92%, continuous positive airway pressure or nasopharyngeal airway use) were recorded.

The study population was divided into two groups; younger than 3 years old and older than 3 years old. All above mentioned variables, interventions and complications were compared between the two groups to assess the risks and safety of PT.

### Statistical analysis

Data were analyzed using SPSS v.28 (IBM Corp., Armonk, NY). Where appropriate, data at each time point were recoded into binary variables (0 = absence, 1 = presence): Patients took either no painkillers or at least one; oxygen saturation was either normal or ≤ 92%; heart rate was either normal or tachycardic; temperature was either normal or indicated a high-grade fever (38.5 degrees Celsius or higher); patients either experienced no bleeding or bleeding; intravenous fluids were either not administered or administered. The first instance of either oral intake or urination were similarly coded, except that once a patient had either eaten or urinated, no new information was coded for these variables across the subsequent time points. Patients were also coded as being discharged or having complications 24 h post-surgery.

To facilitate analyses, data across the five time points were collapsed into single variables. VAS scores at each time point were averaged across all five time points to create a grand mean and were analyzed using independent samples *t*-test. For the remaining variables, if, at least once during the five time points, patients used a painkiller, had oxygen saturation ≤ 92%, were tachycardic, had a high-grade fever, bled, were administered intravenous fluids, had oral intake, or urinated, these variables were coded as 1. Chi-square tests were used to compare these variables between the younger and older groups. We explored whether we had sufficient power to find a large difference between the two groups. For an independent samples *t*-test with two-tailed alpha = . 05, an allocation ratio of 2:1, *d* = 0.80 (Cohen, 1992), and power = 0.80, N = 58 is required. For a chi-square test two-tailed alpha = 0.05, *df* = 1, Cohen’s *w* = 0.50 (Cohen, 1992), and power = 0.80, N = 32 participants are required. Given our total sample of N = 92 children, a sufficient statistical power level was reached.

## Results

Ninety-two children were included in the study, with a mean age of 44.5 ± 21.9 months, (range 12 – 132 months). There were n = 39 females (42%) and n = 53 males (58%). Thirty-five (38%) children were 3-years old or younger and n = 57 (62%) were older than 3 years old. Mean age in the younger group was 25.7 months (SD = 6.9), and 56.1 months (SD = 20.1) in the older group. The number of males and females in each group were not significantly different from each other (males in younger group: n = 23, 66%; males in older group: n = 30, 53%, chi-square(1) = 1.52, *p* = 0.22, Phi = 0.13). (Table 1).

**Table 1:** Demographics of patients three years old or younger and those older than three years old

Variables		Three years old or younger (n = 35)	Older than three years old (n = 57)
Sex	Male (%)	23 (65.7)	30 (52.6)
	Female (%)	12 (34.3)	27 (47.4)
Age (months)	Mean (SD)	25.7 (7.0)	56.1 (20.1)
Weight (kg)	Mean (SD)	12.5 (2.4)	19.4 (8.4)

Across both groups, thirteen (14%) weighed less than 12 kg. In addition to PT, n = 33 (36%) received ventilation tubes.

### Comparison between the groups

Table 2 describes the results of analyses comparing younger patients to older patients on many of the indicators described above. In general, our results indicated similar outcomes across the two groups as no statistically significant differences.

**Table 2:** Results of PITA in children aged younger than or equal to and older than 36 months

Variable	Mean (SD) / N (%)		p-value	Effect size
	Younger group (n = 35)	Older group (n = 57)		
VAS	0.35 (0.47)	0.40 (0.65)	0.74^a^	0.07^c^
Painkiller use	21 (60%)	31 (54%)	0.28^b^	0.06^d^
IV fluids	1 (3%)	5 (9%)	0.27^b^	0.12^d^
Complications withing 24 h	2 (6%)	5 (9%)	0.59^b^	0.06^d^
Oral intake	33 (94%)	55 (96%)	0.30^b^	0.11^d^
Urinated	32 (91%)	50 (88%)	0.58^b^	0.06^d^
*Tachycardia*				
After 2 h	13 (37%)	15 (26%)	0.27^b^	0.11^d^
After 4 h	7 (20%)	10 (18%)	0.77^b^	0.03^d^
After 6 h	10 (29%)	10 (18%)	0.21^b^	0.13^d^
After 8 h	3 (9%)	9 (16%)	0.32^b^	0.10^d^
After 24 h	7 (20%)	7 (12%)	0.32^b^	0.10^d^

^a^ = t-test, ^b^ = chi-square; ^c^ = Cohen’s d; ^d^ = Cohen’s w

There were no significant statistical differences in VAS score between the two groups, 0.35 ± 0.47 in the younger group and 0.4 ± 0.65 in the older group, *p* = 0.74*.* No significant statistical differences were recorded between the groups in the use of painkillers or IV fluid administration. (*p* = 0.28 and *p* = 0.27, respectively). Regarding tachycardia, we found no significant statistical between the two groups at any time point; 2nd time point: *p* = 0.27, 4th: 0.77, 6th:0.21, 8th:0.32, 24th: 0.32. Mean overall oxygen saturation was 97% ± 0.79 in the younger group and older group 98% ± 0.81. Significant differences between the groups were found in the 2nd and 6th time-points (*p* < 0.05 and < 0.01, respectively). However, no significant differences were found at the following timepoints; 4th, 8th, and 24th hour. (Table 3).

**Table 3:** Descriptive and inferential statistics for oxygen saturation for patients three years old or younger and those older than three years old across five time points after surgery

Hours post-surgery	Mean (SD)		*t*-test	*d*
	Three years old or younger (n = 35)	Older than three years old (n = 57)		
2	97.1 (1.3)	97.8 (1.5)	2.1*	0.45
4	97.7 (1.4)	98.3 (1.4)	1.7	0.37
6	97.7 (1.2)	98.4 (0.9)	3.0**	0.65
8	97.9 (1.2)	98.4 (1.3)	1.5	0.34
24	97.9 (1.5)	98.0 (1.4)	0.5	0.10

*p < 0.05. **p < 0.01

By six hours post-surgery, all but four children—two in the younger group (6%) and two in the older group (4%)—had oral intake at least once. These four children did not eat at any point in the subsequent 18 h. Rates of oral intake were not significantly different (chi-square(1) = 1.08, *p* = 0.30, Cohen’s *w* = 0.11). Similarly, by six hours post-surgery all but ten children—three in the younger group (9%) and seven in the older group (12%)—had urinated at least once, with no significant difference between groups (chi-square(1) = 0.31, *p* = 0.58, Cohen’s *w* = 0.06). By eight hours post-surgery, all children had urinated at least once.

### Complications and outcomes

Eighty-five children (92.4%) were discharged after the 24-h follow-up after meeting the discharge criteria. Seven (7.6%) were hospitalized more than 24 h; 4 due to high fever (> 38.5 °C sub-axillary) and 3 due to insufficient oral intake. Of the children in the younger group, n = 2 (5.71%) were not discharged and of the children in the older group, n = 5 (8.77%) were not discharged. The complications were more common in the older group than the younger group without a statistical significance (chi-square(1) = 0.29, *p* = 0.59, Phi = 0.06).

All of the children who had fever were in the older group (n = 4), and underwent fever work-up included chest radiograph and urinalysis. Three were discharged on oral antibiotics due to suspected pneumonia, none of these children had desaturation below 93%. No respiratory complications were found in the younger group. Three children had insufficient oral intake at the 24th hour (2 in the younger group and 1 in the older group). (Table 4).

**Table 4:** Complications among patients three years old or younger and those older than three years old

Complication	Three years old or younger (n = 35)	Older than three years old (n = 57)
Extended admission (more than 24 h) n (%)	2 (5.71%)	5(8.77%)
Fever	0	4
Insufficient oral intake	2	1

All 7 children that needed extended admission were discharged after 48-h of hospitalization and after regaining adequate oral intake, normal temperature, and good general medical condition. No apneas or any major interventions were recorded in any of the groups.

## Discussion

The 2019 Tonsillectomy Clinical Practice Guideline from the American Academy of Otolaryngology–Head and Neck Surgery Foundation [2] advocates inpatient monitoring for all children younger than 3 years old undergoing total tonsillectomy. This recommendation is specially recommended among children with other medical conditions (Syndromic children, obese, or behavioral factors). Several studies published in the literature could have led to this recommendation. In a study of 321 children undergoing total tonsillectomy, 29.2% of those younger than 3-years had a major respiratory complication. However, children with asthma, neuromuscular and congenital heart diseases were included [7]. In another large retrospective study that included 2315 children that underwent total tonsillectomy for OSA, children younger than 3 years old had significantly more postoperative respiratory complications when compared to children aged 3–5 years (9.8% vs. 4.9%). Again, both study groups included children with asthma, obesity, and craniofacial malformation [5]. However, Baijal et al [6]. investigated the rate of perioperative respiratory complications in 880 children undergoing total tonsillectomy, and found that weight < 14 kg was associated with more complications, on the other hand, age was not statistically significant.

PT is a surgical option for treating OSA and potentially chronic tonsillitis. Schmidt et al [10]. compared intracapsular tonsillectomy and traditional tonsillectomy for treating recurrent adeno-tonsillitis or streptococcal pharyngitis. They found no significant difference in the number of post-operative infections in either group (*p* = 0.295). The decrease in recurrent tonsillitis in the PT group was attributed to a disturbance of the bacteriological environment established prior to surgery [10]. In a recent meta-analysis comparing PT to extra-capsular tonsillectomy (traditional tonsillectomy) for treating OSA, PT reduced post-operative pain and bleeding, and facilitated faster return to diet [11]. Another meta-analysis had similar results. A significant improvement in Apnea Hypopnea Index and OSA 18-item questionnaire scores, similar to the traditional tonsillectomy group, was also seen [12].

Despite these benefits of intracapsular tonsillectomy and increased use of this method among otolaryngologists worldwide, it was not mentioned in the 2019 Tonsillectomy Guidelines [2, 13]. The major drawback is a potential regrowth of tonsillar tissue after surgery. However, the rate of symptomatic regrowth of palatine tonsillar tissue is considered low [14, 15].

A recent published meta-analysis and a systematic review, demonstrated lower rates of postoperative complications and a faster return to diet in PT compared to total tonsillectomy [16, 17]. Hence, PT is considered less invasive than total tonsillectomy. We aimed to question the need of inpatient monitoring of children younger than 3 years old undergoing PT. In our results, there were no statistical differences between the younger and older group in the above-mentioned variables; the recovery and return to diet after surgery was similar in the 2 groups, and most importantly, the rate of perioperative complications was not statistically significant. Although statistically significant difference in oxygen saturation were found in the 2nd and 6th time-points; (97.1 vs. 97.8, *p* < 0.05 and 97.7 vs. 98.4, *p* < 0.01, respectively), however, no significant differences were found at the following timepoints; 4th, 8th, and 24th hour. Thus, these changes were not clinically significant, furthermore, respiratory complications were not detected in younger group. These findings are supported by several retrospective studies. Bent et al. [8]. reported the safety of PT in 226 children, and found no statistically significant differences in pain, oral intake or complications, between the younger children (n = 38, less than 3 years old) vs older. Stahl et al. [9] reported no difference in postoperative complications in children younger than 3 years old (n = 48) to older children (n = 59).

We suggest that younger children, without significant comorbidities, undergoing PT for OSA/SDB may undergo ambulatory PT surgery with low risk of complications. However, further larger studies are needed to confirm these findings since the power of our study was limited due small cohort numbers.

As of today, children undergoing PT in publicly-funded hospitals in Israel are admitted for a 24 h surveillance. This conservative approach is a stark deviation from the national Israeli guidelines, which suggested that healthy children older than 2 years old can undergo an ambulatory PT. Hopefully, the results of our study can facilitate decision makers to adopt the national guidelines [4]. Furthermore, ambulatory PT can be mentioned in the next American Academy of Otolaryngology tonsillectomy guidelines.

The current study had some limitations, firstly, the small cohort number in both groups, however, the study was sufficiently powered to detect differences that have d >  = 0.63. The study was underpowered to detect differences that are smaller than that, given our sample size, a power of 0.8, and an alpha of 0.05. Secondly, Monitoring of SpO2 was not continuous. Theoretically, the follow-up could have missed possible respiratory complications, that underwent self-resolution. However, all of these children were admitted and closely observed. The team was instructed to report any change in the clinical status. Furthermore, all the variables collected in the 24th hour, including a heart rate, oxygen saturation and a general physical examination by a physician were used to detect any ongoing respiratory complication. An additional limitation is that all children at our institution underwent a microdebrider assisted PT, although there are multiple methods around the world to perform such procedure.

## Conclusion

The results of this cohort support that children undergoing ambulatory PT may be at low risk of complications, regardless of age.

## Data Availability

The datasets used and/or analyzed during the current study are available from the corresponding author on reasonable request.

## Abbreviations

PT: Partial tonsillectomy
SDB: Sleep disordered breathing
OSA: Obstructive sleep apnea
